# Supplementary figures and images for: Structural optimization of the excavator boom under extreme working conditions using EDEM–ADAMS coupled simulation
Source: PLoS One. 2025 Nov 21;20(11):e0337421. doi: 10.1371/journal.pone.0337421 (PMC12637966; doi:10.1371/journal.pone.0337421)

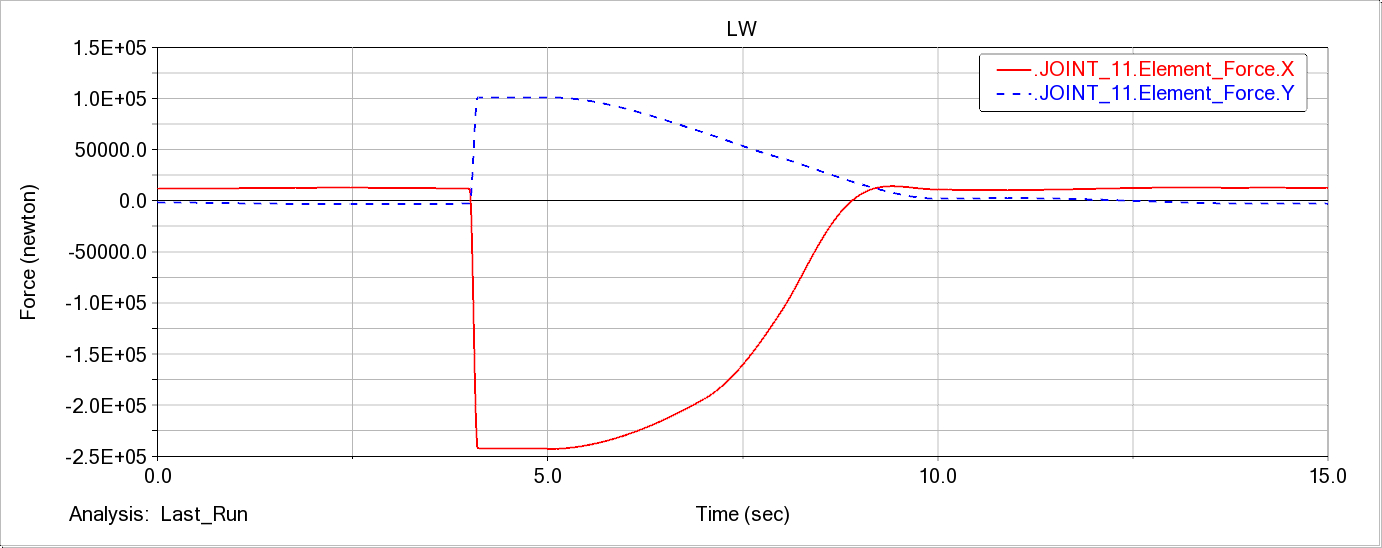

Supplement: S1 File — (ZIP) [file pone.0337421.s001.zip › S1 File. Mechanical data and finite element analysis (FEA) results under the conventional load-spectrum acquisition method/S1 Fig.Force Curve of JOINT_11 under Forward Excavation Conditions.tif]

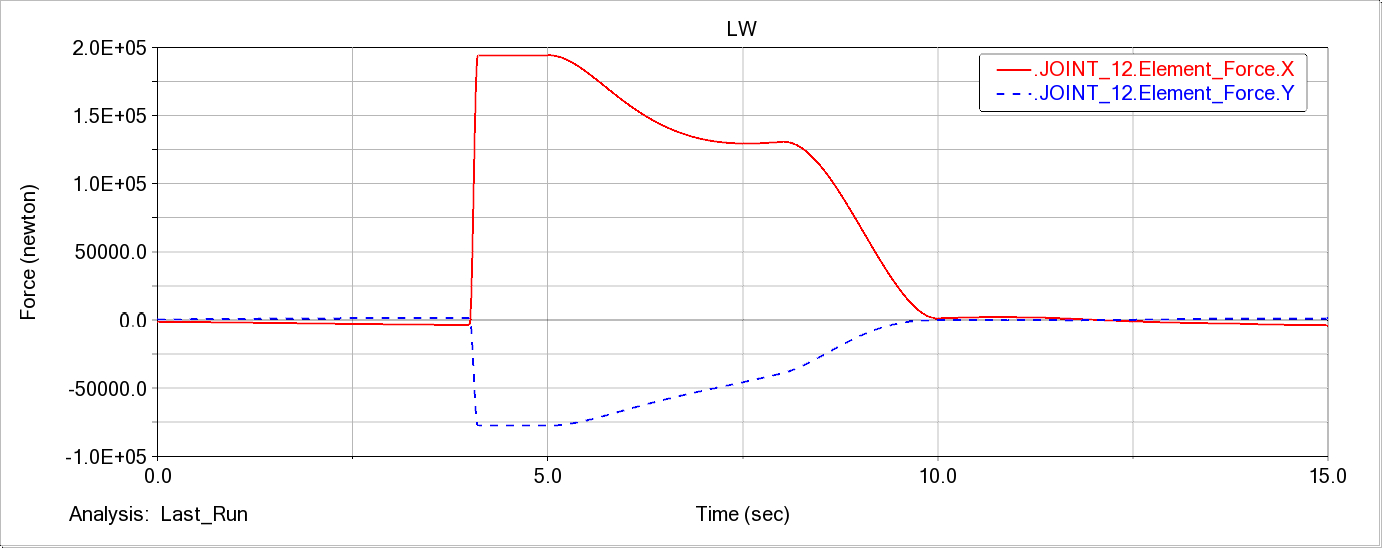

Supplement: S1 File — (ZIP) [file pone.0337421.s001.zip › S1 File. Mechanical data and finite element analysis (FEA) results under the conventional load-spectrum acquisition method/S2 Fig.Force Curve of JOINT_12 under Forward Excavation Conditions.tif]

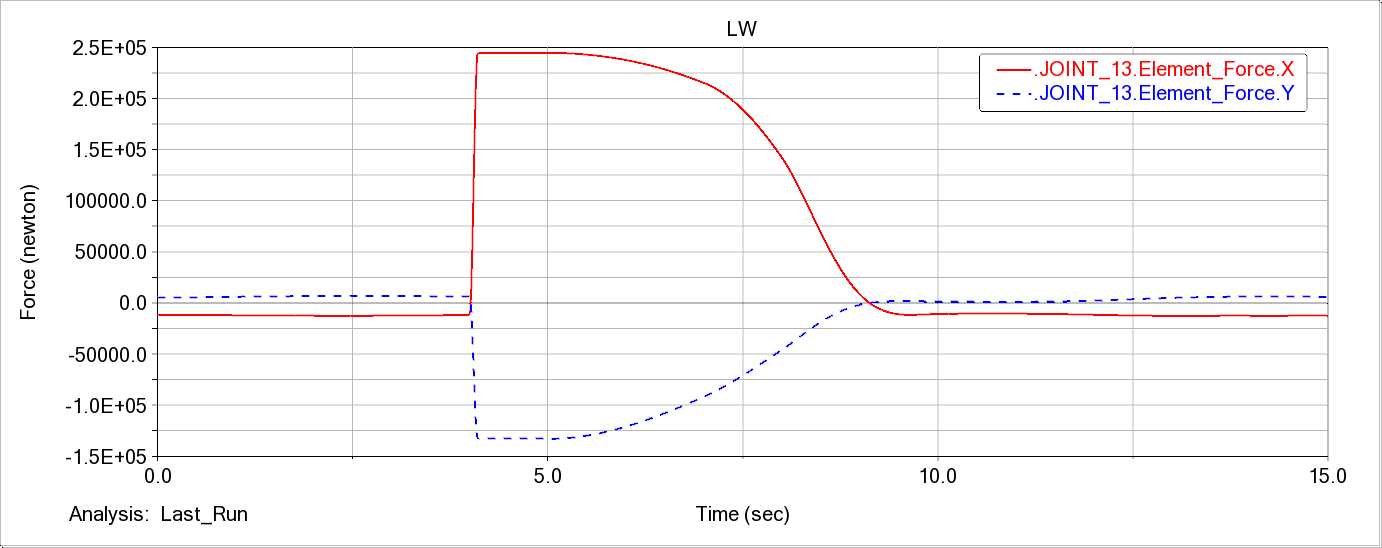

Supplement: S1 File — (ZIP) [file pone.0337421.s001.zip › S1 File. Mechanical data and finite element analysis (FEA) results under the conventional load-spectrum acquisition method/S3 Fig.Force Curve of JOINT_13 under Forward Excavation Conditions.tif]

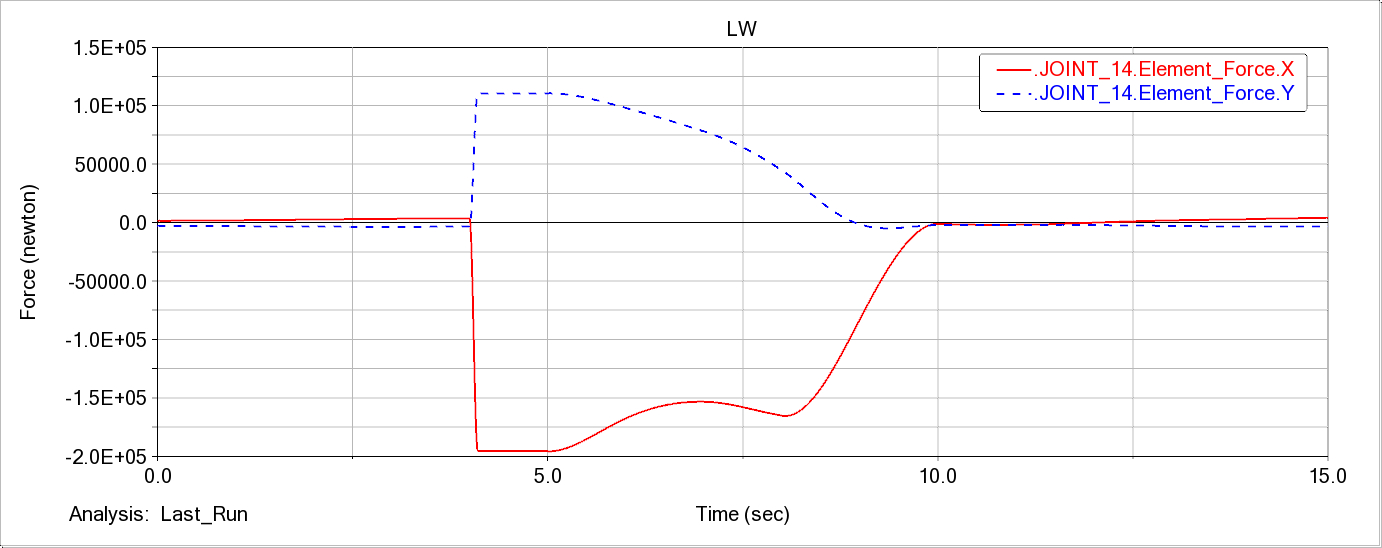

Supplement: S1 File — (ZIP) [file pone.0337421.s001.zip › S1 File. Mechanical data and finite element analysis (FEA) results under the conventional load-spectrum acquisition method/S4 Fig.Force Curve of JOINT_14 under Forward Excavation Conditions.tif]

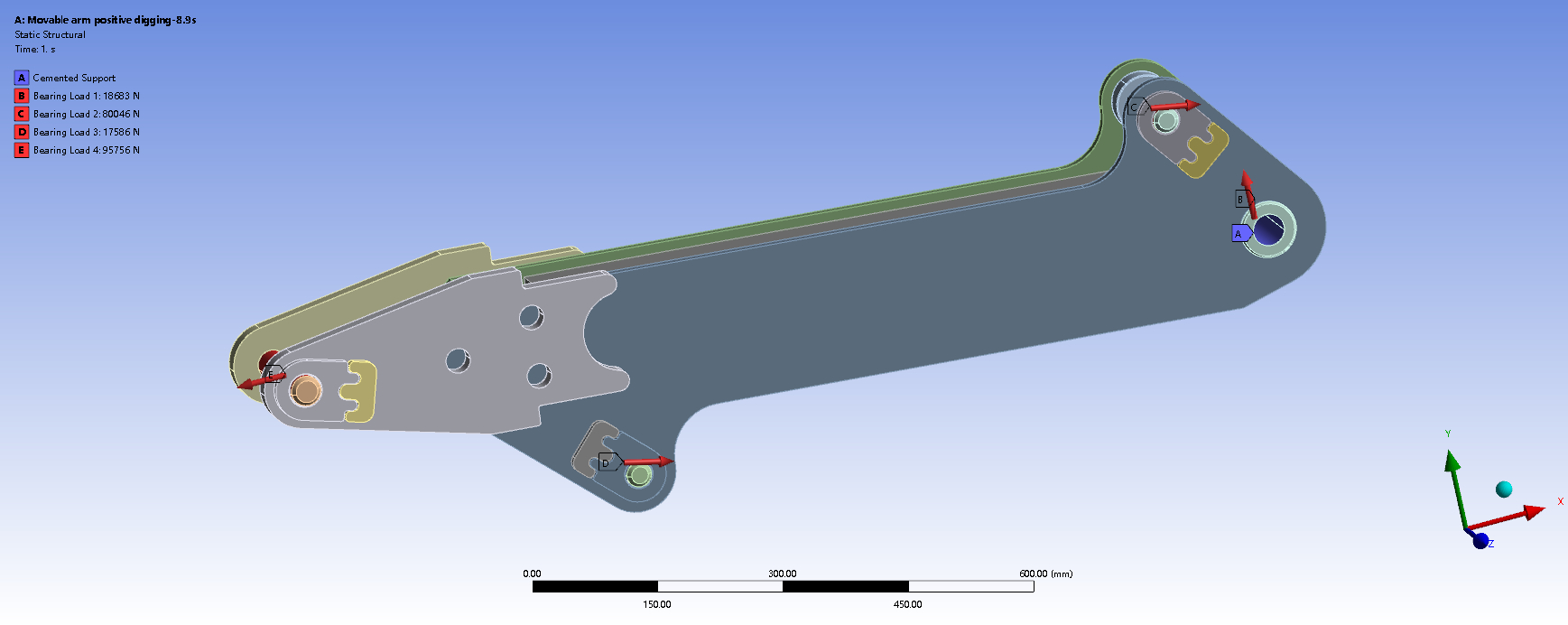

Supplement: S1 File — (ZIP) [file pone.0337421.s001.zip › S1 File. Mechanical data and finite element analysis (FEA) results under the conventional load-spectrum acquisition method/S5 Fig.Load Application for Boom at 8.9 s under Forward Excavation.tif]

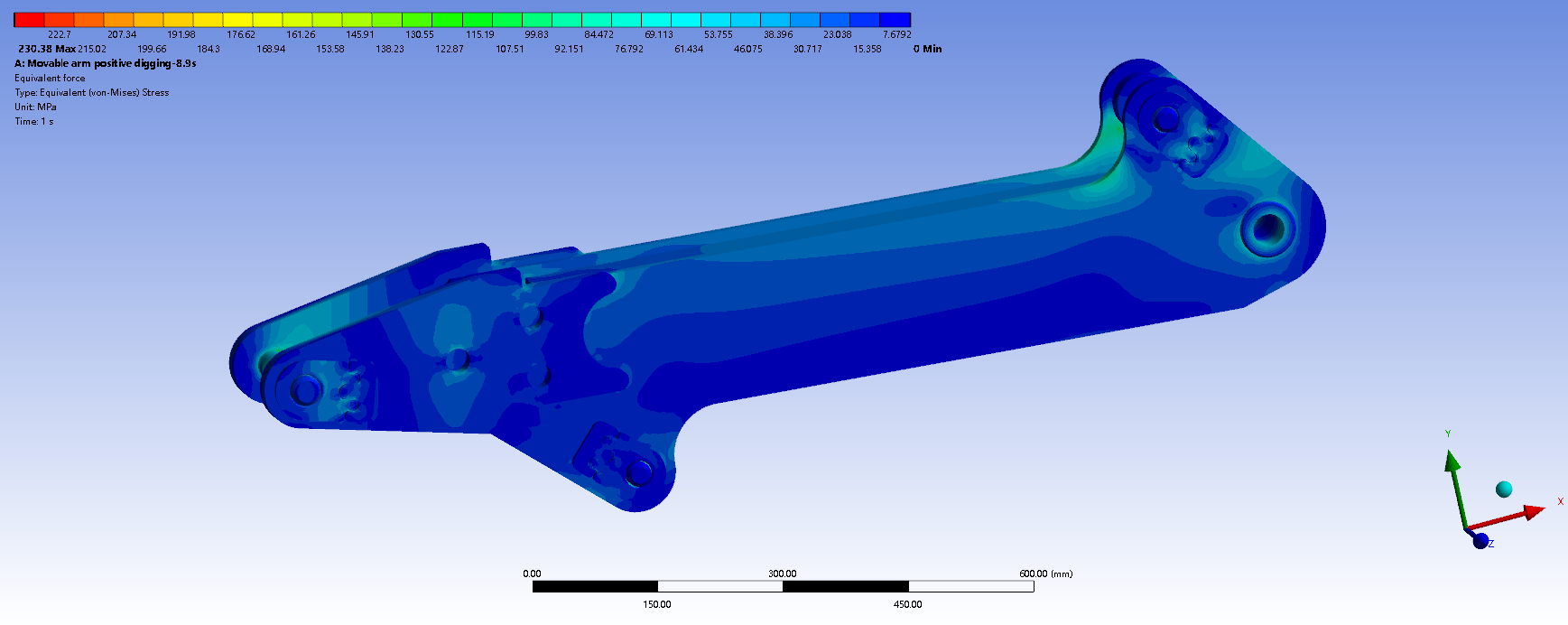

Supplement: S1 File — (ZIP) [file pone.0337421.s001.zip › S1 File. Mechanical data and finite element analysis (FEA) results under the conventional load-spectrum acquisition method/S6 Fig.Equivalent Stress Contour of the Boom at 8.9 s under Forward Excavation.tif]

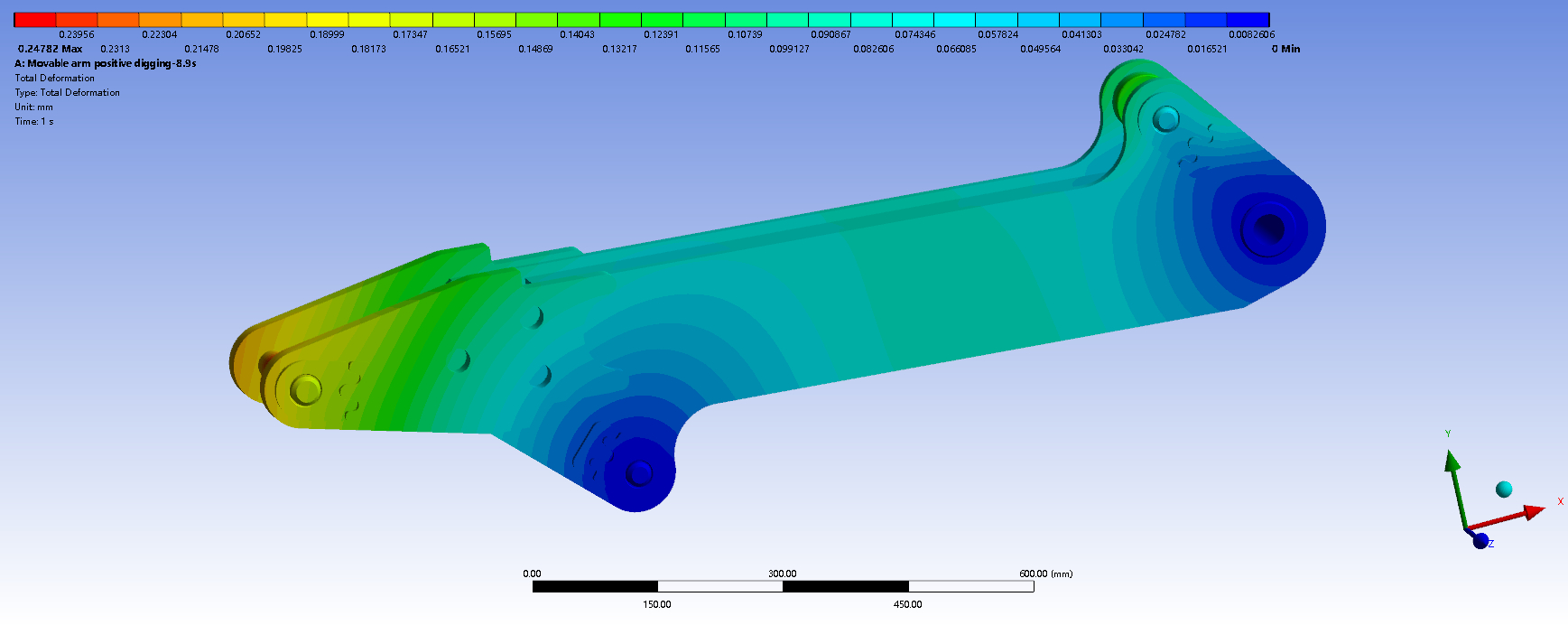

Supplement: S1 File — (ZIP) [file pone.0337421.s001.zip › S1 File. Mechanical data and finite element analysis (FEA) results under the conventional load-spectrum acquisition method/S7 Fig.Total Deformation Contour of the Boom at 8.9 s under Forward Excavation.tif]

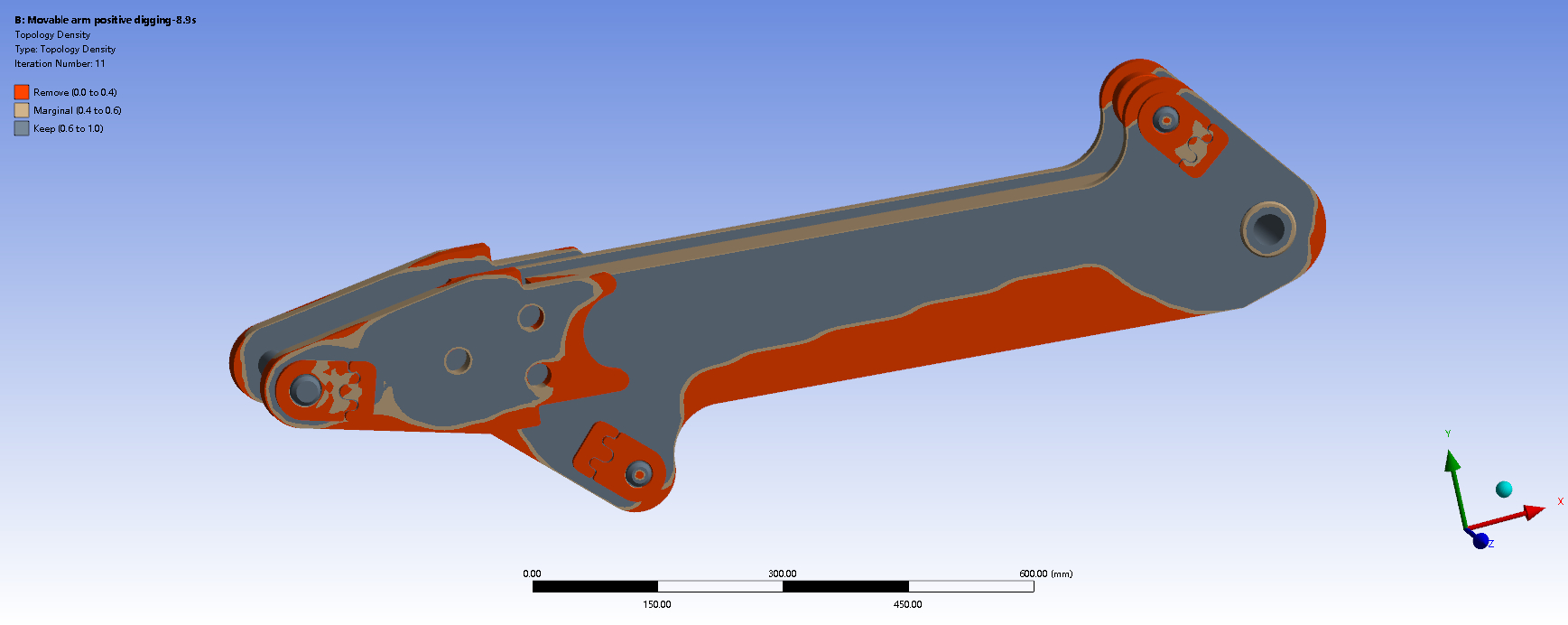

Supplement: S1 File — (ZIP) [file pone.0337421.s001.zip › S1 File. Mechanical data and finite element analysis (FEA) results under the conventional load-spectrum acquisition method/S8 Fig.Load image of boom topology optimization under extreme working conditions.tif]
