# Supplementary material for: Structural optimization of the excavator boom under extreme working conditions using EDEM–ADAMS coupled simulation
Source: PLoS One. 2025 Nov 21;20(11):e0337421. doi: 10.1371/journal.pone.0337421 (PMC12637966; doi:10.1371/journal.pone.0337421)
Supplement: Attachment — Submitted filename: Review Comments.pdf [file pone.0337421.s002.pdf]

Title: Structural Optimization of the Excavator Boom Under Extreme Working Conditions Using EDEM-ADAMS Coupled Simulation

Reviewer Comments:

A kinematic modeling and structural optimization analysis were performed for a Loader-Excavator as a digging transportation device, and attempt is made to improve the performance of boom under critical working condition. The dynamic simulation and working condition identified using the ADAMS software. As well as excavation resistance and hinge-point loads were obtained using EDEM-based excavation simulation. Ultimately achieved the reduction in weight of the boom, deformation and equivalent stresses through structural optimization. Also further reduced equivalent stress and deformation in comparison with the model optimized by conventional method.

I appreciate the efforts made by authors for the current state of the manuscript presented. The abstract is perfectly written. The flow of content presented in the manuscript is perfect and technically correct. Right methodology adopted to justify the title of the manuscript and objectives. But it is very important to justify the following points to improve the quality and readability of the manuscript for the readers of the journal.

Comments for improvement:

- **Section II-A: Prototype Development:** Remove the word "diagram" from Fig. 1**.** Figure caption should be like "Fig. 1 Structural Assembly of the Loader-Excavator".
- **Section II-A: Prototype Development:** Please keep figure for the top view of the Loader-Excavator and change the figure numbers accordingly.
- **Section II-C: Forward kinematics formulation:** In the present study the working range diagram under typical no-load conditions is obtained to verify the forward kinematic solution. But the working range represents the working space through which the bucket tip can reach within in this range. The simulation is also one of the tool to validate the forward kinematics but need to show case the comparison of the input joint angles with bucket tip pose. Recommended to perform comparison of results of simulation with forward kinematics or perform inverse kinematics to validate the forward kinematic model developed for loader-excavator.
- **In the section II-D, it is mentioned that "**Following elimination of redundant degrees of freedom and overconstraints, essential kinematic joints were applied at critical hinge locations" but its methodology is missing to achieve the same. Please add the methodology or justify clearly that how it was achieved.
- **In Fig. 6:** If possible increase the font size of the nomenclatures given to all the joints for better readability.
- **Fig. 7: Scope of work diagram:** Generally, best suitable name for this figure 7 is "Working range diagram" in place of "scope of work diagram".
- **Fig. 7:** Presented working range diagram is overlapping with the shovel front of the loader-excavator. It is also visible in the Fig. 1 and Fig. 7. Might be the presented working range not be achieved as per the visual presentation given in the manuscript. Please justify exactly.

- **Table-6:** Total mass of 780 kg and 1200 kg taken for particle types. Please justify that why different values taken for study?
- **Table-**7: row -1, write $\mathrm{F}_z\ (\mathrm{N})$ in place of Fz (N). Do correction.
- **Table-**8: row-1, unit of Modulus of elasticity should be MPa in place of Mpa. Do correction.
- **Para-1 (Content after the Fig. 16): Presently written last line:** "This kinematic configuration corresponds spatially to the maximum digging depth condition illustrated in Fig. 3.15".
  Here, wrong figure number is written. Please write correct figure number. If might be Fig. 16.
- **Paragraph above Fig. 16 and paragraph below Fig. 17:** To avoid repetition of statements**.**
  [**See Paragraph above Fig. 16:** Notably, the forward excavation condition at t = 8.9 s represents the most critical scenario, exhibiting a peak equivalent stress of 131.42 MPa and a maximum deformation of 0.10777 mm. In contrast, the lateral excavation condition at t = 7.96 s yields the lowest mechanical response, with a peak equivalent stress of 104.13 MPa and a deformation of 0.072722 mm.]
  [**See Paragraph below Fig. 17:** Finite element analysis of the boom under three sets of extreme loading conditions reveals that the equivalent stress and total deformation reach their maximum values at 8.9 s under the forward excavation condition, with respective peaks of 131.42 MPa and 0.10777 mm.
  In contrast, the minimum mechanical response occurs at 7.96 s under the lateral excavation condition, where the equivalent stress and total deformation are 104.13 MPa and 0.07272 mm, respectively.]
- **Section IV: Design Optimization of the Boom and Comparative Validation:** 3rd paragraph, "The boundary condition is defined as a mass retention ratio of 70%". On what basis or consideration, the mass retention ratio taken as 70%? Justify.
- **Section IV-B: Validation and Comparison of the Optimized Design:** In the present case, the conventional optimization approach adopted for comparison of equivalent stress and total deformation of optimized model of boom. There are two conventional approaches for optimization: size optimization and shape optimization. But in the present study, it is not defined that which approach adopted for conventional optimization (i.e. size or shape optimization) for comparison purposes. Also study does not shows any variation in thickness of plates or shapes of the boom. According to the given information in the manuscript, it reflects that only simple FEA performed for the boom and results were compared for equivalent stress and total deformation.
  Please elaborate the justification perfectly and revise the content accordingly.
- **In the conclusion section:** Add the future scope of the presented study. (What kind of extension of the study presented is possible?).
  This will provide motivation and scope to the readers of the journal for their future research work.

Note: Minor Revision is Required. It is required to incorporate/justify the given comments.

****
