# Supplementary material for: Structural optimization of the excavator boom under extreme working conditions using EDEM–ADAMS coupled simulation
Source: PLoS One. 2025 Nov 21;20(11):e0337421. doi: 10.1371/journal.pone.0337421 (PMC12637966; doi:10.1371/journal.pone.0337421)
Supplement: Attachment — Submitted filename: Response to Reviewers.docx [file pone.0337421.s005.docx]

**For research article**

| Response to Reviewer #X Comments | | |
| --- | --- | --- |
| **1. Summary** |  |  |
| Thank you very much for taking the time to review this manuscript(Manuscript Number：PONE-D-25-31404，Title：Structural Optimization of the Excavator Boom Under Extreme Working Conditions Using EDEM–ADAMS Coupled Simulation：Structural optimization design）, your suggestions were invaluable to my article, which I analyzed carefully and revised the content and highlighted in red. | | |
| **2. Point-by-point response to Comments and Suggestions for Reviewer #1** | | |
| **Comments 1:** in D-H transformation matrix (equation 1), in the first row, third column (i.e. element 1,3) there must be “sin.sin” instead of “sin.cos”. | | |
| **Response 1**: *Thank you for pointing this out. I have corrected this technical error by changing the "sin.cos" in Equation (1) to "sin.sin". It appears in Equation (1) on line 112 of the revised manuscript with track changes.* | | |
| **Comments 2:** on page 33, after Fig.16, on the fourth row is written “Fig.3.15” – this figure does not exist. | | |
| **Response 2:** *Thank you for pointing this out. I have corrected this technical error by adding the relevant kinematic analysis verification and changing the correct figure number to "Fig.18". The details are shown in lines 375-381 and 543 of the revised manuscript with track changes.* | | |
| **Comments 3:** *on page 20 is written “scrap-and-dig15” – what does 15 mean?* | | |
| **Response 3:** *Thank you for pointing this out. Due to a typographical error that caused this technical issue, I have corrected it by removing the number 15 and standardizing the professional terminology used in this study. Detailed information is provided on line 395 of the revised manuscript with track changes.* | | |
| **Comments 4:** In the Fig.15. – e) and f), there must be 7,96 s. | | |
| **Response 4:** *Thank you for pointing this out. I have made the corresponding legend modification, which is shown in lines 485 and 529-531 of the revised manuscript with track changes.* | | |
| **Comments 5:** In the Table 3 the kinematic joints can be described more precisely. | | |
| **Response 5:** *Thank you for pointing this out. I have made detailed revisions to the hinge motion pairs in Table 3. To accommodate additional requirements for dynamic simulation verification, an extra table has been added. The original Table 3 has been updated to become Table 4, which now provides precise descriptions of all hinge motion pairs, including their numbers, names, and motion pair types. Detailed information is available in line 300 of the revised manuscript with track changes under Table 4.* | | |
| **Comments 6:** After the formulae (28) - the objective function f(x) can be described in more details with corresponding criteria. | | |
| **Response 6:** *Thank you for your valuable suggestions to improve the paper. I have refined the objective function formula with detailed explanations for each parameter. These enhancements are clearly shown in lines 571-580 of the revised manuscript with track changes. Your expert feedback has greatly enhanced the completeness of my work. I sincerely appreciate all the constructive advice you provided.* | | |
| **3. Point-by-point response to Comments and Suggestions for Reviewer #2** | | |
| **Comments 7:** Certain figures (e.g., FEA stress distributions) do not include numerical scale bars or units, which reduces clarity and makes interpretation more difficult. | | |
| **Response 7:** *Thank you for pointing this out. I have reviewed all images and corrected those that should have been labeled with units or scales but weren't. Detailed information is provided in lines 594-596 of the revised manuscript with track changes. Thank you very much for your valuable feedback, which helped identify technical errors in the article.* | | |
| **Comments 8:** Terminology Consistency – The manuscript uses multiple terms such as “pick-and-dredge,” “digging mechanism,” and “excavating mechanism” interchangeably without clear definitions, which may confuse readers. | | |
| **Response 8:** *Thank you very much for your valuable feedback. I have standardized the technical terminology in the article by uniformly correcting it to "backhoe loader". The detailed modifications are marked in the revised manuscript with track changes at lines 26,29,41,42,99,109,123,133,138,212,221,301,395,406,441,445,560, and 660.* | | |
| **Comments 9:** Kinematics Section Detail – The derivations in the kinematics section are presented in excessive detail for the main text and could be shortened or moved to an appendix to improve readability. | | |
| **Response 9:** *Thank you for pointing this out. I have streamlined the unnecessary derivations in the kinematic analysis while retaining only the essential ones. Detailed information is provided in the revised manuscript with track changes at lines 148-157,164-179, and 186-210.* | | |
| **Comments 10:** No experimental validation – While simulations are comprehensive, there is no physical testing to confirm load cases, deformation magnitudes, or optimization effects. | | |
| **Response 10:** *Thank you very much for your valuable suggestions. While your recommendations demonstrate strong guidance and professional expertise, we currently lack sufficient funding and laboratory conditions to conduct physical experiments for data validation. Our literature review indicates that simulation results closely match experimental data, effectively replicating object motion processes. Detailed information is available in the revised manuscript with track changes (lines 391-393). We will enhance communication with our supervisor to secure additional funding for focused research on novel paper development and related physical experiments. We sincerely appreciate your continued support, as your feedback will guide our ongoing efforts to improve research quality.* | | |
| **Comments 11:** The manuscript requires minor English revision in addition to the correction of typographical errors, such as "Loud image" instead of "Load image" in Fig. 18 caption, emoving the stray number in ‘scrap-and-dig15 transporter, etc. | | |
| **Response 11:** *Thank you for pointing this out. I have corrected the English errors in the article. They appear on lines 278,395,444 and 591 of the revised manuscript with track changes.* | | |
| **4. Point-by-point response to Comments and Suggestions for Reviewer #3** | | |
| **Comments 12:** Section II-A: Prototype Development: Remove the word “diagram” from Fig. 1. Figure caption should be like “Fig. 1 Structural Assembly of the Loader-Excavator”. | | |
| **Response 12:** *Thank you for pointing this out. I have changed the name of Fig 1. It appears on line 99 in the revised manuscript with track changes.* | | |
| **Comments 13:** Section II-A: Prototype Development: Please keep figure for the top view of the Loader-Excavator and change the figure numbers accordingly. | | |
| **Response 13:** *Thank you for pointing this out. I have added the top view and front view with corresponding fig numbers. It is shown in lines 93-99 of the revised manuscript with track changes.* | | |
| **Comments 14:** Section II-C: Forward kinematics formulation: In the present study the working range diagram under typical no-load conditions is obtained to verify the forward kinematic solution. But the working range represents the working space through which the bucket tip can reach within in this range. The simulation is also one of the tool to validate the forward kinematics but need to show case the comparison of the input joint angles with bucket tip pose. Recommended to perform comparison of results of simulation with forward kinematics or perform inverse kinematics to validate the forward kinematic model developed for loader-excavator. | | |
| **Response 14:** *Thank you very much for your valuable suggestions. Your feedback will be crucial in optimizing my article. I have added comparative analysis between input joint angles and bucket tip positions to validate the comparison between kinematic analysis and simulation results. Additionally, I corrected errors in cylinder stroke calculations and angle computations from the kinematic analysis. Finally, by comparing data obtained from both methods, it was shown that the discrepancies between kinematic analysis and simulation analysis were minimal, confirming the reliability of both approaches. Detailed information is available in lines 217-277 and 308-387 of the revised manuscript with track changes. Once again, I appreciate your suggestions as they have significantly enhanced the* | | |
| **Comments 15:** In the section II-D, it is mentioned that “Following elimination of redundant degrees of freedom and overconstraints, essential kinematic joints were applied at critical hinge locations” but its methodology is missing to achieve the same. Please add the methodology or justify clearly that how it was achieved. | | |
| **Response 15:** *Thank you for pointing this out. The method for eliminating redundant degrees of freedom has been supplemented. Details are shown in lines 285-295 of the revised manuscript with track changes.* | | |
| **Comments 16:** In Fig. 6: If possible increase the font size of the nomenclatures given to all the joints for better readability. | | |
| **Response 16:** *Thank you for pointing this out. I have made the image modifications shown in Fig 9, lines 298-299 of the revised manuscript with track changes.* | | |
| **Comments 17:** Fig. 7: Scope of work diagram: Generally, best suitable name for this figure 7 is “Working range diagram” in place of “scope of work diagram”. | | |
| **Response 17:** *Thank you for pointing this out. I have revised the legend, which is shown in line 310 of the revised manuscript with track changes.* | | |
| **Comments 18:** Fig. 7: Presented working range diagram is overlapping with the shovel front of the loader-excavator. It is also visible in the Fig. 1 and Fig. 7. Might be the presented working range not be achieved as per the visual presentation given in the manuscript. Please justify exactly. | | |
| **Response 18:** *Thank you for pointing this out. The presence of material baffles may introduce visual inaccuracies in the working range diagram. However, during actual operation, the mining device did not exhibit motion interference with other components. I have revised the diagram by adding detailed zoom-in views to demonstrate simulation results from multiple angles. As shown in Fig 1, no motion interference occurred. Detailed information is provided in lines 309-310 (Fig 10) and lines 92-99 (Fig 1) of the revised manuscript with track changes.* | | |
| **Comments 19:** Table-6: Total mass of 780 kg and 1200 kg taken for particle types. Please justify that why different values taken for study? | | |
| **Response 19:** *Thank you for pointing this out. Based on existing literature, I have supplemented the rationale for particle type configuration and corrected errors in the original article's particle mass measurement results. Detailed information is provided in lines 413-418 of the revised manuscript with track changes and in Table 8 on line 426.* | | |
| **Comments 20:** Table-7: row -1, write F_z (N) in place of Fz (N). Do correction. | | |
| **Response 20:** *Thank you for pointing this out. I have corrected the corresponding technical error, which appears in line 492 of table 9, row 1, of the revised manuscript with track changes.* | | |
| **Comments 21:** Table-8: row-1, unit of Modulus of elasticity should be MPa in place of Mpa. Do correction. | | |
| **Response 21:** *Thank you for pointing this out. I have corrected the corresponding English error, which appears in line 499 of table 10, row 1, of the revised manuscript with track changes.* | | |
| **Comments 22:** Para-1 (Content after the Fig. 16): Presently written last line: “This kinematic configuration corresponds spatially to the maximum digging depth condition illustrated in Fig. 3.15”. Here, wrong figure number is written. Please write correct figure number. If might be Fig. 16. | | |
| **Response 22:** *Thank you for pointing this out. I have corrected this technical error by adding the relevant kinematic analysis verification and changing the correct figure number to "Fig.18". The details are shown in lines 375-381 and 543 of the revised manuscript with track changes.* | | |
| **Comments 23:** Paragraph above Fig. 16 and paragraph below Fig. 17: To avoid repetition of statements. [See Paragraph above Fig. 16: Notably, the forward excavation condition at t = 8.9 s represents the most critical scenario, exhibiting a peak equivalent stress of 131.42 MPa and a maximum deformation of 0.10777 mm. In contrast, the lateral excavation condition at t = 7.96 s yields the lowest mechanical response, with a peak equivalent stress of 104.13 MPa and a deformation of 0.072722 mm.] [See Paragraph below Fig. 17: Finite element analysis of the boom under three sets of extreme loading conditions reveals that the equivalent stress and total deformation reach their maximum values at 8.9 s under the forward excavation condition, with respective peaks of 131.42 MPa and 0.10777 mm. In contrast, the minimum mechanical response occurs at 7.96 s under the lateral excavation condition, where the equivalent stress and total deformation are 104.13 MPa and 0.07272 mm, respectively.] | | |
| **Response 23:** *Thank you for pointing this out. I have removed the duplicate statement. Details are shown in lines 551-558 of the revised manuscript with track changes.* | | |
| **Comments 24:** Section IV: Design Optimization of the Boom and Comparative Validation: 3rd paragraph, “The boundary condition is defined as a mass retention ratio of 70%”. On what basis or consideration, the mass retention ratio taken as 70%? Justify. | | |
| **Response 24:** *Thank you for pointing this out. I have added a basis and consideration for a 70% mass retention rate based on the suggestion and existing literature. Detailed information is shown in lines 583-585 of the revised manuscript with track changes.* | | |
| **Comments 25:** Section IV-B: Validation and Comparison of the Optimized Design: In the present case, the conventional optimization approach adopted for comparison of equivalent stress and total deformation of optimized model of boom. There are two conventional approaches for optimization: size optimization and shape optimization. But in the present study, it is not defined that which approach adopted for conventional optimization (i.e. size or shape optimization) for comparison purposes. Also study does not shows any variation in thickness of plates or shapes of the boom. According to the given information in the manuscript, it reflects that only simple FEA performed for the boom and results were compared for equivalent stress and total deformation. Please elaborate the justification perfectly and revise the content accordingly. | | |
| **Response 25:** *Thank you for pointing this out. The comparison between the optimization method described in this article and existing methods is based on different load spectrum data acquisition approaches. I have added corresponding detailed descriptions and revised relevant content. Detailed information is shown in lines 610-640 of the revised manuscript with track changes.* | | |
| **Comments 26:** In the conclusion section: Add the future scope of the presented study. (What kind of extension of the study presented is possible?). This will provide motivation and scope to the readers of the journal for their future research work. | | |
| **Response 26:** *Thank you very much for your valuable feedback. Your suggestions will help make my article more complete and professional. I have added a future outlook section to the article. Detailed information is available in lines 662-675 of the revised manuscript with track changes.* | | |
