# Supplementary material for: Structural optimization of the excavator boom under extreme working conditions using EDEM–ADAMS coupled simulation
Source: PLoS One. 2025 Nov 21;20(11):e0337421. doi: 10.1371/journal.pone.0337421 (PMC12637966; doi:10.1371/journal.pone.0337421)
Supplement: Attachment — Submitted filename: Reviewer Decision.docx [file pone.0337421.s004.docx]

**Title:** Structural Optimization of the Excavator Boom Under Extreme Working Conditions Using EDEM-ADAMS Coupled Simulation

**Reviewer Comments:**

All corrections have been incorporated in the updated manuscript and now no further correction is required in the current state of the manuscript.

**Decision:** Accepted the manuscript for publication.
